# Peroral endoscopic myotomy for a residual Zenker’s diverticulum following endoscopic myotomy

**DOI:** 10.1016/j.vgie.2021.10.001

**Published:** 2021-10-30

**Authors:** Qais Dawod, Sanad Dawod, David Carr-Locke, Reem Z. Sharaiha, Kartik Sampath

**Affiliations:** Division of Gastroenterology & Hepatology, NewYork-Presbyterian Hospital and Weill Cornell Medical Center, New York, New York

**Keywords:** Z-POEM, Zenker's peroral endoscopic myotomy

## Abstract

Video 1 Peroral endoscopic myotomy for a residual Zenker’s diverticulum following endoscopic myotomy.

Peroral endoscopic myotomy for a residual Zenker’s diverticulum following endoscopic myotomy.

Symptomatic Zenker’s diverticulum can be associated with significant morbidity. Zenker’s diverticulum can persist after flexible endoscopic myotomy. Several endoscopic and surgical techniques can be considered, but it is unclear which interventions are feasible and appropriate for residual Zenker’s diverticulum.

A 49-year-old man with a medical history of GERD presented with a symptomatic Zenker’s diverticulum. The patient noted 2 years of intermittent dysphagia to solids and at times to liquids. A rigid endoscopy was performed by otolaryngology, with planned cricopharyngeal myotomy. However, despite multiple attempts, the septum was not visualized, and the procedure was aborted. The patient was referred for flexible endoscopic myotomy.

The patient was placed in the supine position and underwent general anesthesia (Video 1, available online at www.giejournal.org). The endoscope was passed under direct visual guidance, and a small posterior Zenker's diverticulum (Fig. 1) was noted. An orogastric tube was placed to orient the diverticulum and expose the cricopharyngeal septum. At the center of the septum, a perpendicular incision into the submucosal space was made using an endoscopic submucosal dissection IT2 knife (Endocut Q Current, Olympus, Center Valley, Pa, USA) (Table 1). Myotomy was performed through the septum muscle fibers and extended to the apex of the diverticulum (Fig. 2). After myotomy, 2 Hemoclips (ConMed, Utica, NY, USA) were applied to close the mucosotomy.

**Figure 1:**
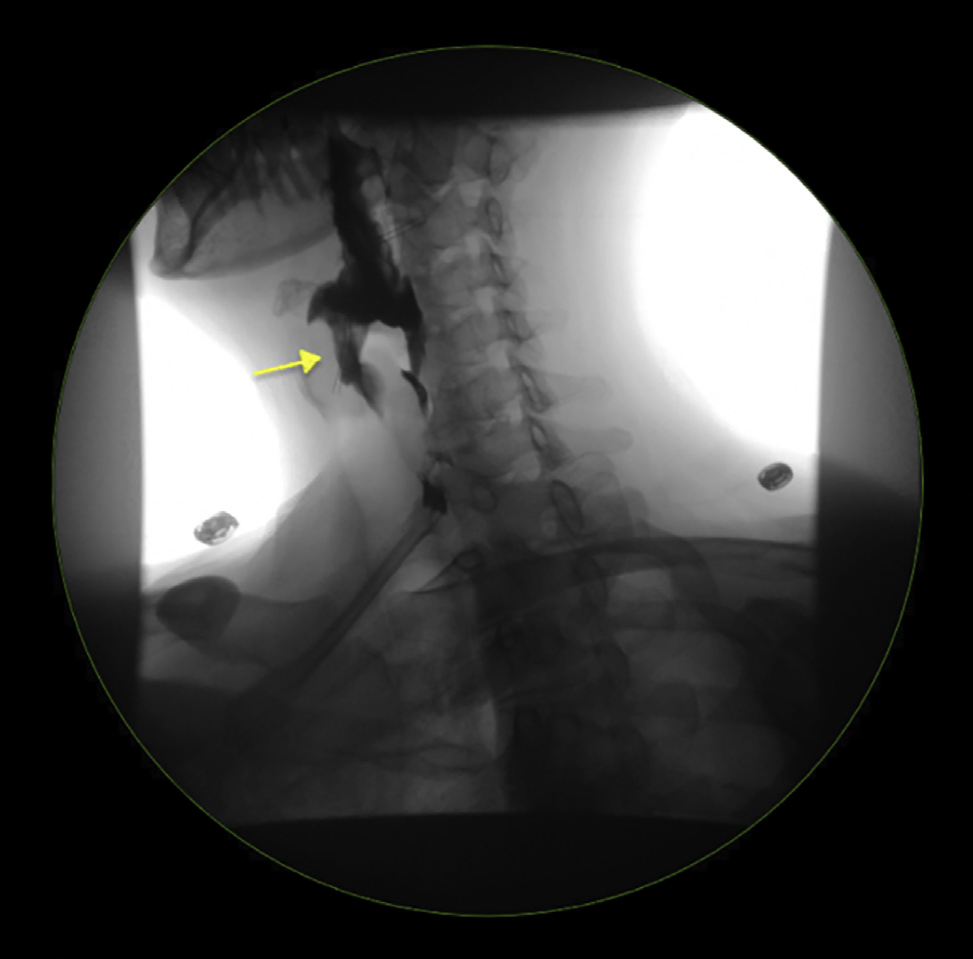
Esophagram showing the Zenker’s diverticulum.

**Table 1:** Electrosurgical generator unit type and settings for the first and second procedures

Mode	ENDO CUT Q	FORCED COAG	DRY CUT	SOFT COAG
Effect	3	2	3	5
Max watts	NA	50	80	80
Cut duration/interval	1	NA	NA	NA

*NA,* Not available.

**Figure 2:**
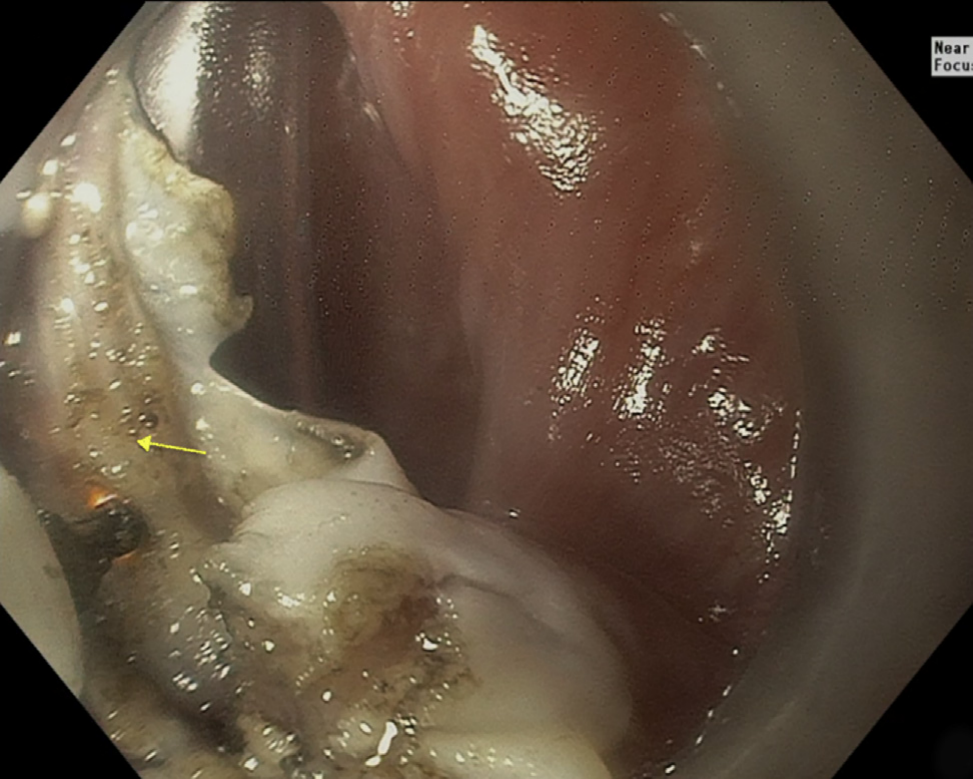
Circumferential muscle fiber myotomy.

The patient was admitted for observation for 1 night after the procedure, and no antibiotics were given. The patient noted immediate symptom relief. Two months later, recurrent dysphagia was reported, and symptoms progressed. Repeat esophagram revealed a residual Zenker’s diverticulum. Endoscopy was planned with potential repeat myotomy with the tunneled Zenker's peroral endoscopic myotomy (Z-POEM) technique.

The patient was again placed in the supine position and under general anesthesia for the second procedure. The endoscope was passed under direct visual guidance, and a residual Zenker's diverticulum with a pronounced septum was found. A scar was noted from the prior myotomy. The decision was made to perform Z-POEM. The center of the septum was injected with a methylene blue/saline solution (Fig. 3), and a good lift was noted at the prior septotomy scar site. A transverse mucosotomy along the septum was performed with an endoscopic submucosal dissection Dualknife (Endocut Q Current, Olympus) (Table 1). Intermittent injection of saline/methylene blue solution was continued into the Zenker's diverticulum and the esophageal submucosal sides of the cricopharyngeal septum, and submucosal dissection was performed. After dissection, the full septum was exposed. Septum myotomy was performed (Endocut Q Current), extending directly to the visualized apex of the diverticulum (Fig. 4). The mucosotomy was closed using 5 duraclips (Fig. 5), and no antibiotics were given. After the Z-POEM, the patient was discharged home. At the 2-month follow-up, the patient noted marked symptom relief.

**Figure 3:**
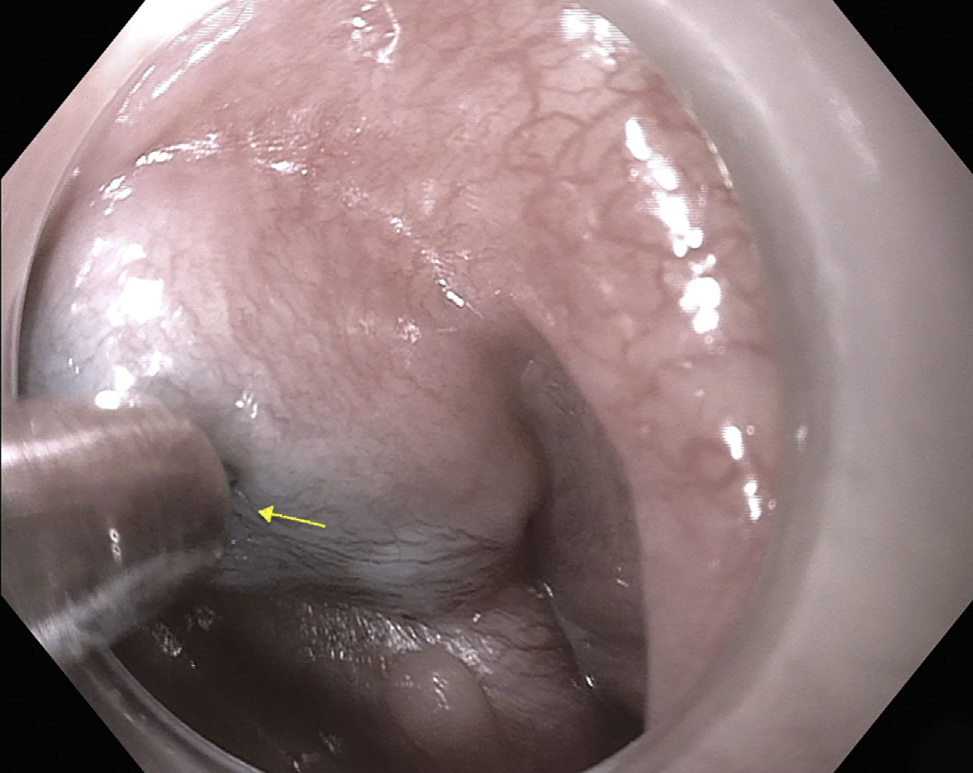
Center of septum injected with methylene blue saline solution.

**Figure 4:**
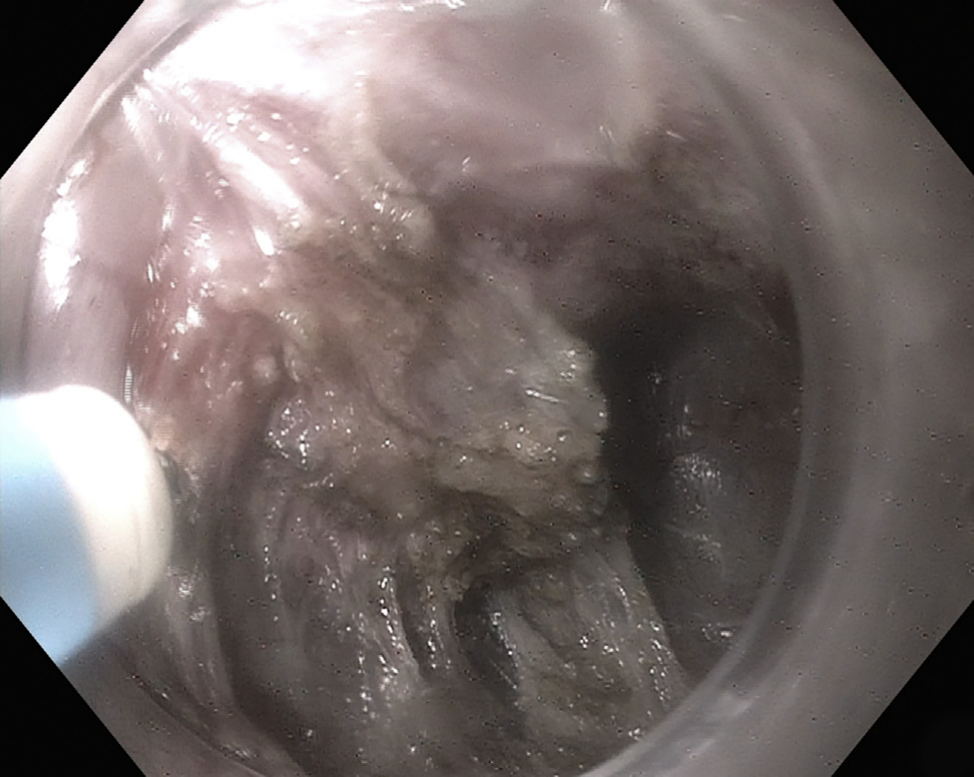
After complete submucosal dissection, circumferential myotomy was performed.

**Figure 5:**
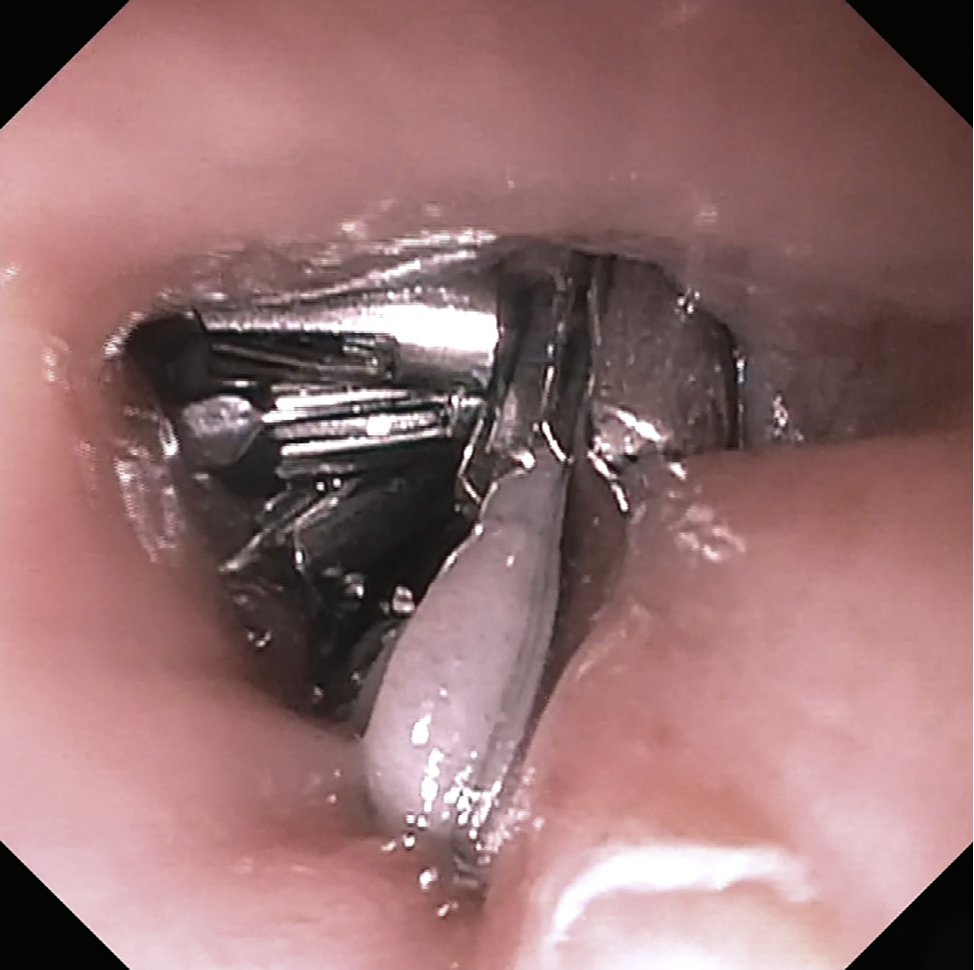
Mucosotomy closed with hemostatic clips.

Flexible endoscopic myotomy is an effective intervention for treating Zenker’s diverticulum.^1^^,^^2^ Repeat endoscopic assessment is important in recurrent/persistent Zenker’s diverticulum. Z-POEM is a viable rescue intervention because it may safely permit more aggressive division of the septum.^3^^,^^4^

## Disclosure

*Dr Carr-Locke is a consultant for Boston Scientific; receives royalties from Steris and Telemed; and is a patent holder for Valentx, Ergogrip, and Screwire. Dr Sharaiha is a consultant for Cook, Boston Scientific, and Olympus. All other authors disclosed no financial relationships.*

## Supplementary data

Video 1 Peroral endoscopic myotomy for a residual Zenker’s diverticulum following endoscopic myotomy.
